# Supplementary material for: Relationship between COVID-19 Mortality, Hospital Beds, and Primary Care by Italian Regions: A Lesson for the Future
Source: J Clin Med. 2022 Jul 19;11(14):4196. doi: 10.3390/jcm11144196 (PMC9315865; doi:10.3390/jcm11144196)
Supplement: Supplementary file 1 [file jcm-11-04196-s001.zip › jcm-1809807-supplementary.pdf]

FIGURE LEGENDS

Figure S1. Relationship between COVID-19 deaths (x 10000 inhabitants) and the percentage of over 65 years old subjects *per* Italian regions. No association (r^2=0.8393, p=0.266) between COVID-19 deaths (x 10000 inhabitants) and the percentage of over 65 years old subjects was found. The line shows the linear regression value obtained by the multivariate analysis.

Figure S 2. Relationship between COVID-19 deaths (x 10000 inhabitants) and Life expectancy *per* Italian regions. No association (r^2=0.8292, p=0.427) between COVID-19 deaths (x 10000 inhabitants) and the Life Expectancy was found. The line shows the linear regression value obtained by the multivariate analysis.

Figure S3. **Relationship between COVID-19 deaths (x 10000 inhabitants) and Aging Index *per* Italian regions.** No association (r^2=0.8292, p=0.427) between COVID-19 deaths (x 10000 inhabitants) and the Aging Index was found. The line shows the linear regression value obtained by the multivariate analysis.

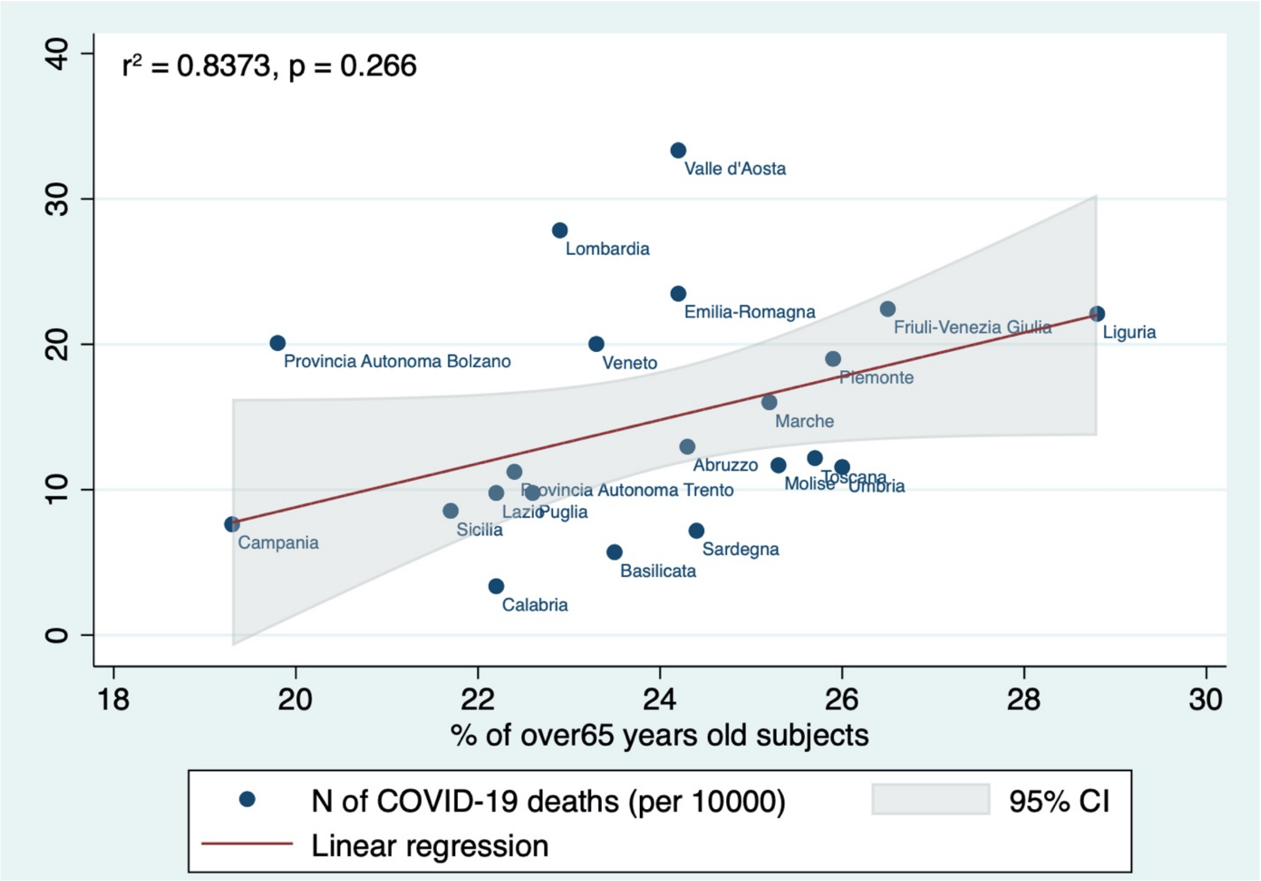

Figure S1

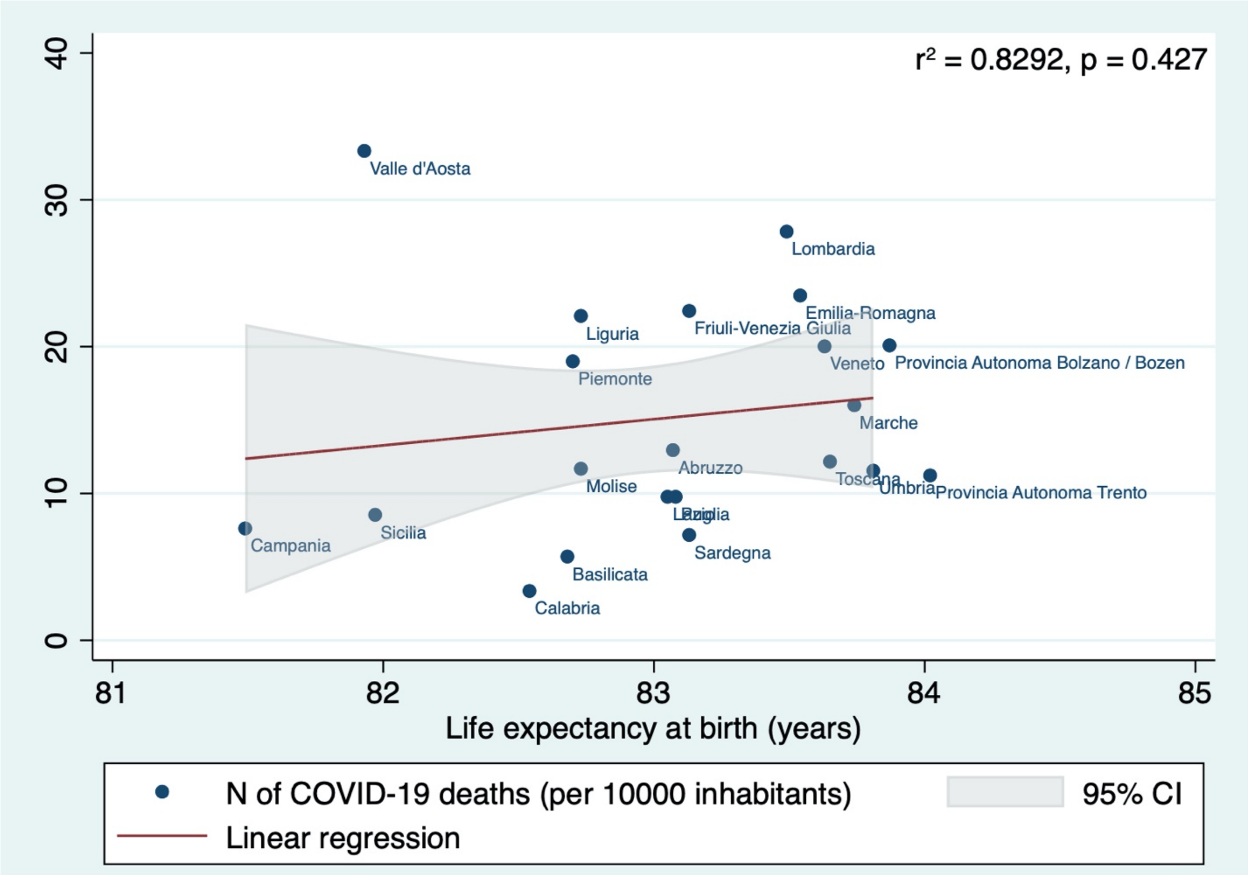

Figure S2

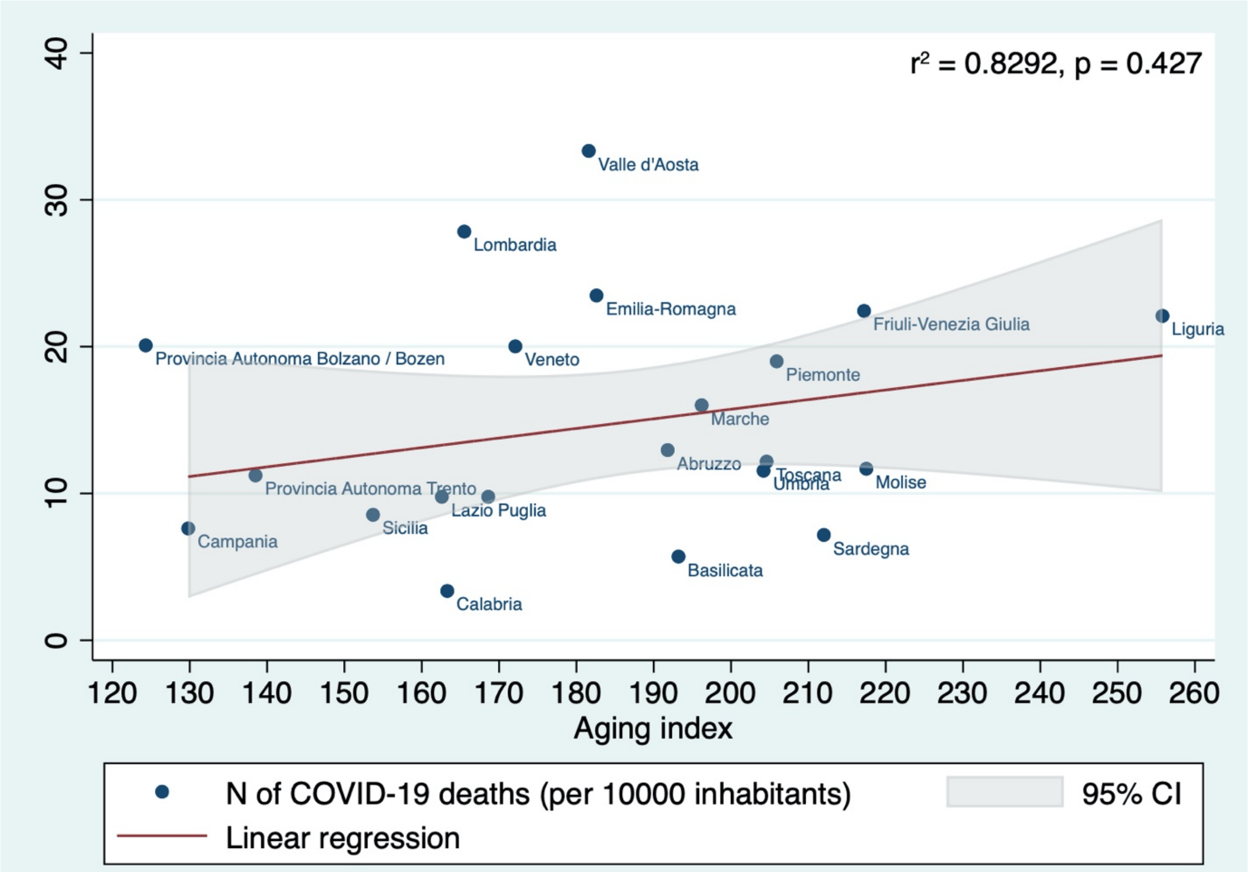

r² = 0.8292, p = 0.427
Valle d'Aosta
Lombardia
Emilia-Romagna
Friuli-Venezia Giulia
Liguria
Provincia Autonoma Bolzano / Bozen
Veneto
Piemonte
Marche
Abruzzo
Toscana
Umbria
Molise
Provincia Autonoma Trento
Lazio
Puglia
Sicilia
Campania
Sardegna
Basilicata
Calabria
0
10
20
30
40
120
130
140
150
160
170
180
190
200
210
220
230
240
250
260
Aging index
N of COVID-19 deaths (per 10000 inhabitants)
95% CI
Linear regression

Figure S3

Checklist of information that should be included in new reports of global health estimates

Item #	Checklist item	Reported on page #
**Objectives and funding**		
1	Define the indicator(s), populations (including age, sex, and geographic entities), and time period(s) for which estimates were made.	3,4
2	List the funding sources for the work.	7
**Data Inputs**		
For all data inputs from multiple sources that are synthesized as part of the study:		
3	Describe how the data were identified and how the data were accessed.	3,4
4	Specify the inclusion and exclusion criteria. Identify all ad-hoc exclusions.	3,4
5	Provide information on all included data sources and their main characteristics. For each data source used, report reference information or contact name/institution, population represented, data collection method, year(s) of data collection, sex and age range, diagnostic criteria or measurement method, and sample size, as relevant.	3,4
6	Identify and describe any categories of input data that have potentially important biases (e.g., based on characteristics listed in item 5).	3
For data inputs that contribute to the analysis but were not synthesized as part of the study:		
7	Describe and give sources for any other data inputs.	3
For all data inputs:		
8	Provide all data inputs in a file format from which data can be efficiently extracted (e.g., a spreadsheet rather than a PDF), including all relevant meta-data listed in item 5. For any data inputs that cannot be shared because of ethical or legal reasons, such as third-party ownership, provide a contact name or the name of the institution that retains the right to the data.	7
**Data analysis**		
9	Provide a conceptual overview of the data analysis method. A diagram may be helpful.	4
10	Provide a detailed description of all steps of the analysis, including mathematical formulae. This description should cover, as relevant, data cleaning, data pre-processing, data adjustments and weighting of data sources, and mathematical or statistical model(s).	4
11	Describe how candidate models were evaluated and how the final model(s) were selected.	4,5
12	Provide the results of an evaluation of model performance, if done, as well as the results of any relevant sensitivity analysis.	NA
13	Describe methods for calculating uncertainty of the estimates. State which sources of uncertainty were, and were not, accounted for in the uncertainty analysis.	4
14	State how analytic or statistical source code used to generate estimates can be accessed.	4
**Results and Discussion**		
15	Provide published estimates in a file format from which data can be efficiently extracted.	Table 1
16	Report a quantitative measure of the uncertainty of the estimates (e.g. uncertainty intervals).	4,5<br>Table 2, Fig 1-6
17	Interpret results in light of existing evidence. If updating a previous set of estimates, describe the reasons for changes in estimates.	5-7
18	Discuss limitations of the estimates. Include a discussion of any modelling assumptions or data limitations that affect interpretation of the estimates.	7

This checklist should be used in conjunction with the GATHER statement and Explanation and Elaboration document, found on gather-statement.org
